# Role of serum periostin level as profibrotic marker in conjunction with renal resistivity index and shear wave elastography in predicting renal fibrosis in diabetic nephropathy

**DOI:** 10.1186/s12882-025-04612-3

**Published:** 2025-11-28

**Authors:** Heba Mahmoud Ibrahim, Suldan A. Ali, Tarek A. Ramzy, Nehal H. Elsaid, Amr Mohamed Shaker

**Affiliations:** 1 Departments of Internal Medicine and Endocrinology, Kasr Al Aini Hospital, Cairo University, Cairo, Egypt; 2 Departments of Internal Medicine and Nephrology, Kasr Al Aini Hospital, Cairo University, Cairo, Egypt; 3 Department of Chemical Pathology, Kasr Al Aini Hospital, Cairo University, Cairo, Egypt

**Keywords:** DKD, Renal fibrosis, Serum periostin, RRI, PSWE

## Abstract

**Background:**

Renal fibrosis is crucial to be detected early in diabetic kidney disease (DKD). Serum periostin is a biomarker for the early detection of fibrosis in DKD. Moreover, shear wave elastography (PSWE) and the renal resistivity index (RRI) are widely used to evaluate renal fibrosis. We aimed to evaluate periostin’s role as an indicator for detecting renal fibrosis in the early stages of DKD, alongside RRI and PSWE.

**Methods:**

Seventy-two type 2 diabetes mellitus patients were subdivided into four groups dependent on glomerular filtration rate (GFR) categories (G1–G4), and eighteen healthy participants were enrolled. Creatinine, HbA1c, eGFR, albumin-creatinine ratio (ACR), and serum periostin were measured. RRI and PSWE values were recorded.

**Results:**

Periostin levels increased significantly with DKD progression. Periostin’s diagnostic performance of cutoff value, which was 39.92 ng/mL for early detection of DKD, with 97.2% sensitivity and 100% specificity. Both RRI and PSWE values increased significantly with DKD progression. RRI and PSWE cutoff values were 0.5 and 1.52 kPa, respectively, with sensitivity of 83.3% and specificity of 100% for RRI, and for PSWE values demonstrated a sensitivity of 80.6%, and specificity of 100%. Strong positive correlations were observed between periostin levels and both RRI and PSWE values (*p* < 0.001).

**Conclusions:**

Periostin, RRI and PSWE provide valuable insight into the combined use of biochemical and imaging markers for early detection of renal fibrosis in DKD monitoring of DKD progression.

**Trial registration no:**

MS-132-2024.

## Introduction

Diabetic kidney disease (DKD) representing a major diabetes’ microvascular issue and a foremost reason of chronic kidney disease (CKD). The progressive decline in renal function associated with DKD is induced via the renal fibrosis’ accumulation, characterized by glomerulosclerosis and tubulointerstitial fibrosis. Early detection and management of renal fibrosis are critical for preventing progression to ESRD [1, 2].

Periostin is crucial biomarker in tissue remodeling. Elevated levels of periostin have been strongly associated with increased accumulation of fibrous material in the kidneys, making it a valuable biomarker in DKD. As DKD progresses, overexpression of periostin correlates with the progression of tubulointerstitial fibrosis and glomerulosclerosis [3, 4].

The renal resistivity index (RRI), a Doppler ultrasound-derived parameter, measures intrarenal vascular resistance and is a non-invasive method to assess renal hemodynamics, elevated RRI values indicate increased intrarenal resistance, often attributable to underlying renal fibrosis and vascular damage. The ability of RRI to differentiate between various stages of DKD highlights its diagnostic utility in clinical practice [5].Point shear wave elastography (PSWE) is an advanced imaging technique that validates tissue fibrosis, making it a reliable tool for staging the disease in DKD patients [6].

## Patients and methods

This research was conducted on 72 diabetic patients, randomly selected from the inpatient units of the internal medicine departments and during follow-up visits at the outpatient clinic of Kasr Al Aini Hospital between March 2024 and December2024). The patients were categorized according to KDIGO 2022 for DKD (albuminuria (urine albumin-to-creatinine ratio ≥ 30 mg/g]), and/or decreased eGFR (< 60 mL/min/1.73 m²) that persists for ≥ 3 months.) Patients were classified into four groups according to GFR (group 1 GFR > 90 ml/min/1.73m2, group 2 GFR 60–89 ml/min/1.73m2, group3 GFR 30–59 ml/min/1.73m2, group 4 15–29 mL/min) and Eighteen healthy individuals were included as a control group.

The study included type II diabetes mellitus patients of both sex within age 30–65 years and excluded patients more than 65 years or below 30 years, BMI > 40 Kg/m^2^, GFR < 15 (calculated by CKD -EPI equation), Malignancy, chronic infections, Pregnant and lactating ladies, liver and cardiovascular disease patients, Solitary kidney, renal artery stenosis or any renal disease other than DKD. The study was approved by the ethical of KasrAlAiny Hospitals (approval code: march 2024 MS-132-2024).

Informed written consent was obtained from all cases and control participants. All cases underwent a detailed physical examination and history taking, including body mass index (BMI), diabetes duration, and medication records. The assessments included glycated hemoglobin (%), estimated glomerular filtration rate (eGFR), creatinine (mg/dL), using the 2021 CKD-EPI formula, and a spot urine test to measure the albumin-to-creatinine ratio (ACR) (mg/g). Additionally, periostin (ng/mL) was measured using a human periostin ELISA kit (Bioassay Technology Laboratory, Cat. No: E3226Hu).

### Imaging techniques

#### Renal Doppler ultrasound

A Philips ultrasound system with a curved transducer (C5-1) was used to assess the renal artery resistivity index (RI). Pulsed wave examination and color Doppler ultrasound were performed on the renal arteries at an angle of 30–35° and with color gain set at 50%. RI was determined as the mean of three diverse segments of intrarenal arteries (lower, mid, and upper poles). RI was calculated via ultrasound utilizing the equation:

$$\:RI=\frac{\mathrm{P}\mathrm{e}\mathrm{a}\mathrm{k}\:\mathrm{S}\mathrm{y}\mathrm{s}\mathrm{t}\mathrm{o}\mathrm{l}\mathrm{i}\mathrm{c}\:\mathrm{V}\mathrm{e}\mathrm{l}\mathrm{o}\mathrm{c}\mathrm{i}\mathrm{t}\mathrm{y}\:\left(\mathrm{P}\mathrm{S}\mathrm{V}\right)-\mathrm{E}\mathrm{n}\mathrm{d}\:\mathrm{D}\mathrm{i}\mathrm{a}\mathrm{s}\mathrm{t}\mathrm{o}\mathrm{l}\mathrm{i}\mathrm{c}\:\mathrm{V}\mathrm{e}\mathrm{l}\mathrm{o}\mathrm{c}\mathrm{i}\mathrm{t}\mathrm{y}\:\left(\mathrm{E}\mathrm{D}\mathrm{V}\right)}{\mathrm{P}\mathrm{e}\mathrm{a}\mathrm{k}\:\mathrm{S}\mathrm{y}\mathrm{s}\mathrm{t}\mathrm{o}\mathrm{l}\mathrm{i}\mathrm{c}\:\mathrm{V}\mathrm{e}\mathrm{l}\mathrm{o}\mathrm{c}\mathrm{i}\mathrm{t}\mathrm{y}\:\left(\mathrm{P}\mathrm{S}\mathrm{V}\right)}$$

#### Point shear wave elastography (PSWE)

B-mode ultrasound employed a curved transducer (C5-1) to locate the kidney and identify regions of interest (ROI). The elastography mode was then activated. Measurements were obtained from multiple kidney regions, including the medulla and cortex. Each measurement was repeated triplicate for each kidney to confirm reproducibility. Tissue stiffness was quantified in kPa.

#### Statistical methods

It was performed using Statistical Package for Social Sciences software (SPSS) version 28. Variables were presented as mean, standard deviation, range, numbers and percentage. Chi square and one way analysis of variance (ANOVA) were used for comparison [7, 8]. Spearmans correlation test was considered [9]. P-value ≤ 0.05 was considered significant.

## Results

Table 1, there were statistically significant variations between the diverse stages of DKD cases and the healthy control group in mean age, BMI, duration of diabetes, creatinine, ACR, HbA1c, and eGFR.(*p* < 0.001). Additionally, significant variations were noted in serum periostin levels across DKD categories compared to the healthy control group (*p* < 0.001), suggesting that periostin levels progressively increase with DKD severity.

The imaging parameters, illustrated in Table 1 show statistically significant differences in mean RRI and PSWE values in both kidneys between DKD categories and the healthy control group (*p* < 0.001). A significant raise in these parameters was noted with advancing GFR categories, underscoring their potential as markers of disease progression.

**Fig. 1:**
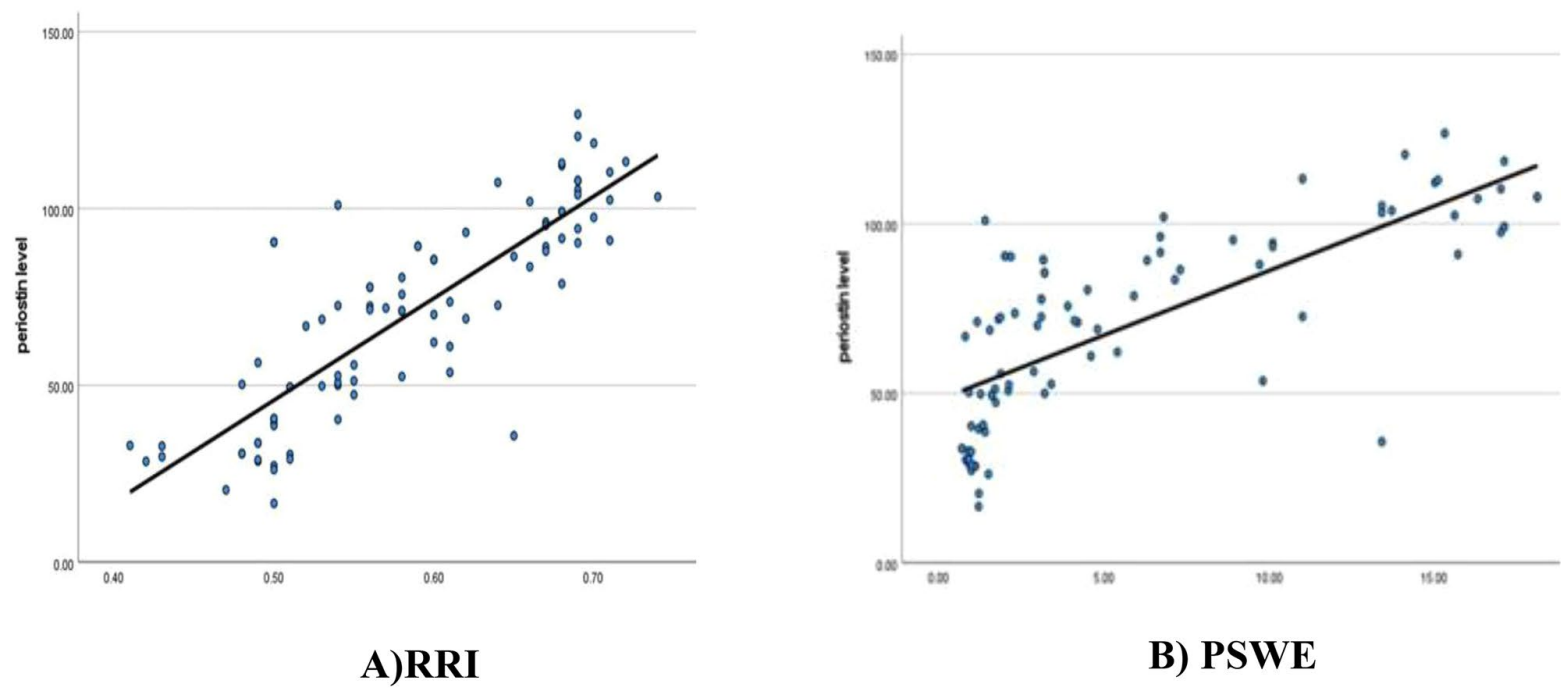
(**A**, **B**): Correlation of serum periostin levels with renal resistivity index (RRI) and point shear wave elastography (PSWE) in DKD

Table 2 demonstrates positive correlations between periostin levels and clinical/laboratory parameters, including the, creatinine, diabetes’ period, ACR, HbA1c and both RRI and PSWE values (*p* < 0.001) (Fig. 1A and B), and negative correlation with eGFR, emphasizing periostin’s reliability as a biomarker for worsening kidney function and fibrosis in DKD. Also, it highlights significant correlations between RI, PSWE, and other clinical/laboratory variables Age, diabetes duration, BMI, ACR and HbA1c positively correlated with renal resistivity and stiffness, indicating worsening renal function with higher values and negative correlations with eGFR. These findings demonstrate that decreased kidney function is associated with increased renal resistivity and stiffness.All correlations were statistically significant (*p* < 0.001), indicating that higher resistivity indices are strongly linked to elevated kidney stiffness, reflecting greater fibrosis and renal impairment.

**Fig. 2:**
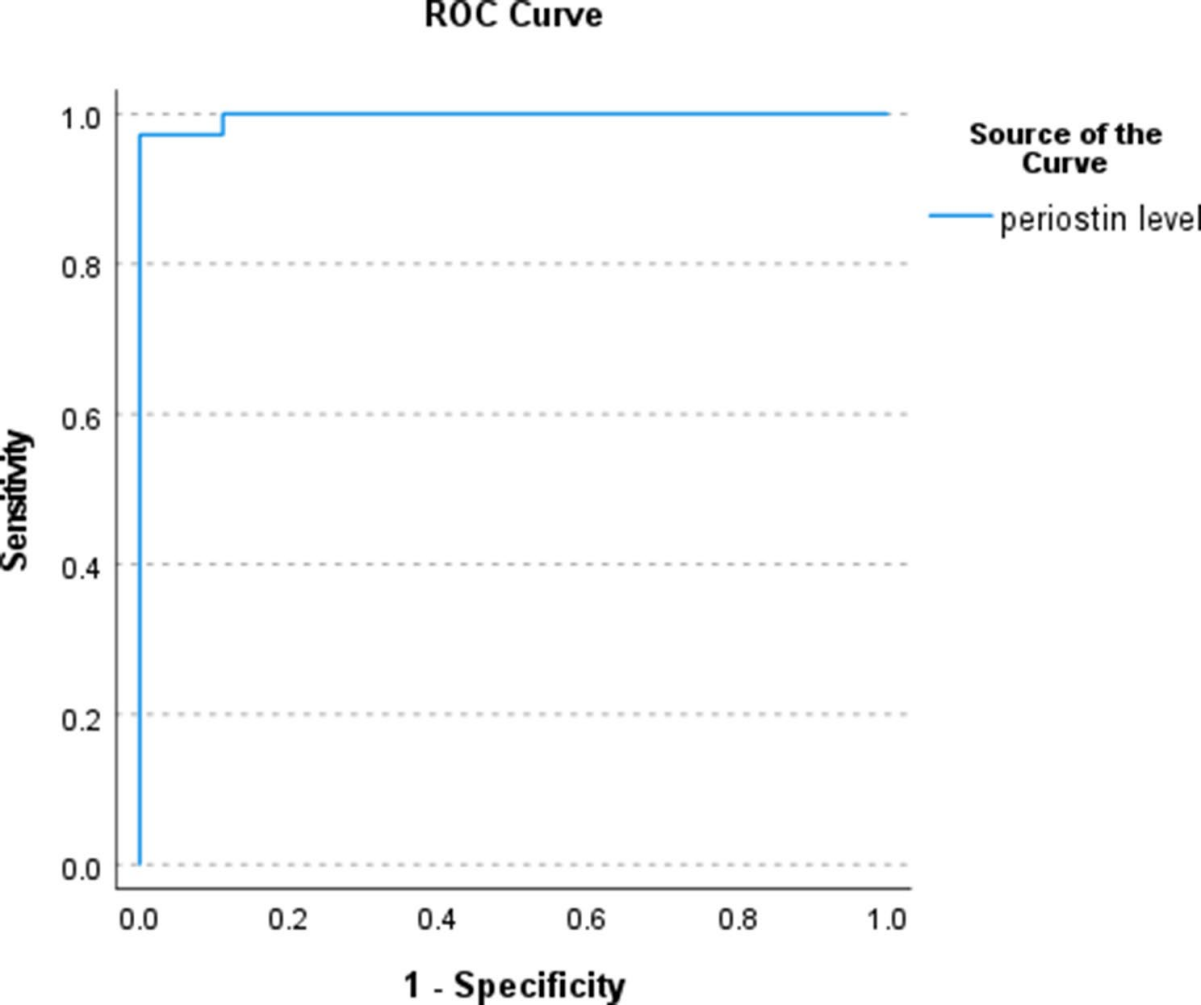
ROC Curve analysis of serum periostin

Table 3 showed periostin exhibited excellent diagnostic performance, with a specificity of 100%, sensitivity of 97.2%, and an AUC of 0.997. The high AUC indicates that periostin can accurately differentiate patients with and without early fibrosis. The identified cutoff value of 39.92 ng/mL provides a practical threshold for clinical use, enabling early detection and timely intervention (Fig. 2).

For RRI, values increased significantly across DKD categories, with a specificity of 100%, a sensitivity of 83.3%, an AUC of 0.951, and a cutoff value of 0.515. PSWE demonstrated a specificity of 100%, a sensitivity of 80.6%, an AUC of 0.927, and a cutoff value of 1.52 kPa. The high AUC values suggest that combining RI and PSWE can effectively distinguish patients with early fibrosis. Table 4 Multivariate logistic regression analysis adjusting for age, BMI, and diabetes duration was done to confirm that periostin independently predicts severe fibrosis.

**Table 1:** Demographic and laboratory investigations of studied subjects

	Normal		G1 DN		G2 DN		G3 DN		G4 DN		*P* value
	Mean	SD	Mean	SD	Mean	SD	Mean	SD	Mean	SD	
Age	51.17	12.22	47.28	10.27	51.94	9.61	57.06	15.52	61.89	7.51	0.002
Duration of diabetes (years)	.	.	5.50	2.57	13.33	5.39	17.72	4.13	24.17	7.19	< 0.001*
BMI (kg/m^2^)	22.28	1.49	26.11	2.37	28.00	2.85	28.69	2.92	29.89	3.45	< 0.001*
creatinine	0.70	0.18	0.69	0.17	1.10	0.41	1.59	0.49	3.39	0.50	< 0.001*
ACR (mg\g)	13.17	3.68	27.00	9.68	83.72	13.24	292.39	31.12	729.78	153.98	< 0.001*
HbA1C	4.53	0.38	8.14	1.25	8.89	2.05	9s.69	1.31	10.83	1.62	< 0.001*
eGFR (mL/min)	111.33	37.22	116.06	12.51	83.61	4.63	48.83	10.45	15.44	0.62	< 0.001*
Periostin	29.92	5.47	51.54	7.75	71.28	11.04	90.11	7.08	107.72	8.81	< 0.001*
Average RRI of both kidney	0.48	0.03	0.54	0.04	0.58	0.03	0.63	0.06	0.69	0.02	< 0.001*
Average PSWE (kPa) of both kidney	1.02	0.22	2.31	2.03	3.85	2.67	6.14	3.15	15.56	1.89	< 0.001*

Data are presented as mean ± SD.* Significant P value < 0.05. Data are presented as mean ± SD or frequency (%). * Significant P value < 0.05. BMI: Body mass index, ACR: albumin to creatinine ratio, HbA1C: glycated hemoglobin, eGFR: estimated glomerular filtrate rate. DN: diabetic nephropathy. RRI: renal resistivity index, Point shear wave elastography, PSWE

**Table 2:** Correlation between (Periostin Levels, RRI and PSWE and clinical parameters)

		Periostin level	RRI of both kidney	Average PSWE (Kpa) of both kidney
Age	r	0.379	0.421	0.347
	P	< 0.001*	< 0.001*	< 0.001*
Duration of diabetes (years)	r	0.795	0.700	0.660
	P	< 0.001*	< 0.001*	< 0.001*
BMI (kg/m^2^)	r	0.424	0.412	0.515
	P	< 0.001*	< 0.001*	< 0.001*
HbA1C (%)	r	0.742	0.761	0.722
	P	< 0.001*	< 0.001*	< 0.001*
ACR (mg\g)	r	0.941	0.888	0.872
	P	< 0.001*	< 0.001*	< 0.001*
eGFR (mL/min)	r	-0.841	-0.796	-0.778
	P	< 0.001*	< 0.001*	< 0.001*
Average RRI of both kidney	r	0.854		0.905
	P	< 0.001*		**< 0.001**
Average PSWE (kPa) of both kidney	r	0.835	0.905	
	P	< 0.001*	**< 0.001**	

r: correlation coefficient. * Significant P value < 0.05. RRI: renal resistivity index of the right kidney, ElastPQ: elastography, BMI: Body mass index, ACR: albumin to creatinine ratio, HbA1C: glycated hemoglobin, eGFR: estimated glomerular filtrate rate. RRI: renal resistivity index of the right kidney, point shear wave elastography (PSWE)

**Table 3:** Diagnostic performance of Periostin level and sonographic parameters for early detection of fibrosis in DKD:

	Area under the curve	*P* value	95% Confidence interval				
			Lower bound	Upper bound	Cut off	Sensitivity %	Specificity %
Periostin	0.997	< 0.001	0.989	1.005	39.92	97.2	100
RRI	0.951	< 0.001	0.897	1.004	0.515	83.3	100
PSWE (kPa)	0.927	< 0.001	0.858	0.997	1.52	80.6	100

**Table 4:** Multivariate regression analysis adjusting for age, BMI, and diabetes duration would strengthen the conclusion that Periostin independently predicts severe fibrosis

		*P* value	OR	95% C.I.	
				Lower	Upper
Severe fibrosis	Periostin level	0.002	1.366	1.118	1.668
	Age	0.308	1.060	0.947	1.187
	Duration of diabetes (years)	0.368	1.102	0.892	1.362
	BMI	0.641	1.123	0.691	1.825

## Discussion

DKD is extensively studied worldwide, which places a significant burden on healthcare systems and adversely impacts patients’ quality of life. Accurate validation and early detection of renal fibrosis are crucial for prompt diagnosis and management of DN, which can slow disease progression and improve patient outcomes.

The current study highlights the potential of periostin, in conjunction with RRI and PSWE, as a reliable multiparametric approach for the assessment of renal fibrosis in diabetic kidney disease (DKD). Our study addressed the effects of various risk factors for DKD, including age, diabetes duration, BMI, HbA1c, creatinine, and eGFR, across different disease stages.

Afkarian et al. noted that older age is linked with a greater risk of developing DN and that the progression of kidney disorders is more prevalent among older diabetic patients [10]. Our findings showed that BMI remained relatively stable across all DN stages, slightly increasing from normal to G1 DN and stability through G4 DN. This observation aligns with Kramer et al., who indicated that while obesity is a known risk factor for DKD development, BMI alone may not significantly change as the disease progresses [11].

Our findings indicated a clear upward trend in diabetes duration from G1 to G4 DKD, suggesting that longer diabetes duration is linked to more advanced DKD stages. This is in line with a study by Tekalign et al., conducted on 614 diabetic patients, which confirmed that individuals with a longer history of diabetes (averaging 16 years) are at greater risk of developing more severe forms of DKD [12].

Furthermore, our study demonstrated a significant increase in serum creatinine levels and a corresponding decrease in eGFR as DN progressed from G1 to G4. ACR levels progressively increased from normal subjects to the highest levels observed in G4 DKD. Perkovic et al. emphasized that elevated creatinine levels, decreased eGFR, and persistent albuminuria are strongly associated with advanced DKD stages, underscoring their critical role in guiding therapeutic decisions for diabetic patients [13].

In our study, periostin levels significantly increased with advancing stages of DKD. The mean periostin levels were 29.92 ng/mL in the normal group, progressively rising to 107.72 ng/mL in G4 DKD (*p* < 0.001). Moreover, a strong positive association was identified between periostin levels and ACR (*r* = 0.941, *p* < 0.001), while a strong negative association was noted between periostin levels and eGFR (*r* = -0.841, *p* < 0.001). These findings suggest that periostin levels rise in response to increasing fibrotic activity within the kidneys, reflecting DKD progression.

These observations are consistent with previous studies highlighting periostin’s role as a biomarker in fibrotic processes in diabetic patients. For example, El-Dawla et al. investigated the value of serum periostin as a prospective marker of disorder progression in diabetic patients. In their study, They discovered that group with macroalbuminuria had a marked increase in periostin [14].

Similarly, Abdel Ghafar et al. conducted a cross-sectional study involving 137 T2DM patients that revealed urinary periostin levels, measured using ELISA, were significantly greater in DN groups compared to the normoalbuminuric T2DM group [15].

Furthermore, Satirapoj et al. analyzed urinary periostin levels in 328 adult T2DM cases and 30 healthy controls. They found that elevated urinary periostin levels in diabetic patients were significantly associated with increased albuminuria and decreased eGFR, corroborating the results of our study [16].

In this study, strong positive correlations were identified between periostin levels and key clinical parameters, including the diabetes duration (*r* = 0.795), BMI (*r* = 0.424), and HbA1C (*r* = 0.742). These findings align with the outcomes of previous research. For instance, a study by Luo et al. reported significant associations between periostin levels, BMI, and HbA1C, further supporting periostin’s potential as a multifaceted marker that reflects fibrosis and various aspects of metabolic dysfunction in DKD [17].

Our study’s RRI demonstrated significant diagnostic potential for detecting renal fibrosis in DKD. A strong positive correlation was noted between RRI and ACR, while a strong negative correlation was noted with eGFR. The mean RRI values for the left kidney significantly elevated from 0.69 ± 0.02 in G4 DKD (*p* < 0.001).

These results align with a cross-sectional comparison study by Kuttancheri et al., which included 114 participants (56 non-diabetic and 58 diabetic cases) who underwent renal Doppler and renal biopsy for resistivity index assessment. Their study revealed significantly higher renal resistivity index values in diabetic patients. The research noticed a strong positive correlation between RRI and all histopathological indices in both non-diabetic and diabetic groups, further confirming the utility of RRI as a reliable indicator of renal fibrosis and dysfunction [18].

Additionally, a cross-sectional study by Ali et al. analyzed 82 type 2 diabetic patients. Their findings revealed that patients with diabetic nephropathy had higher RRI values compared to the control group. Furthermore, RRI values showed a significant positive correlation with albuminuria, supporting utilizing RRI as a vital marker for renal dysfunction in diabetic nephropathy [19].

In our study, PSWE (kPa) demonstrated strong diagnostic potential for detecting renal fibrosis in DKD. The PSWE values for both kidneys increased significantly with the progression of DKD. The mean PSWE values increased to 15.56 ± 1.89 kPa in G4 DKD (*p* < 0.001). These results affirm the utility of PSWE as a reliable, non-invasive tool for assessing renal stiffness and detecting fibrotic changes in DKD. Moreover, PSWE showed positive correlations with ACR and creatinine levels and a negative correlation with eGFR, further supporting its diagnostic function in the progression of renal fibrosis.

Our findings align with the literature by Mo et al., who performed a meta-analysis to assess the diagnostic accuracy of PSWE for staging renal fibrosis. The analysis included 405 patients across multiple studies and demonstrated that PSWE is a highly accurate, non-invasive method for assessing renal fibrosis [20]. Similar to our study, a meta-analysis by Cao et al. aimed to validate the diagnostic performance of PSWE for staging renal fibrosis in CKD dependent on renal biopsy pathology. They concluded that PSWE is a reliable approach for diagnosing renal fibrosis [21]. Additionally, another study by Chen et al. used a nomogram based on PSWE to assess renal fibrosis in CKD cases. This study, involving 162 CKD patients, found that combining PSWE with clinical features improved diagnostic accuracy for staging renal fibrosis [22].

Furthermore, our results coincide with those of Yuksekkaya et al., who studied 108 cases with T2DKD and 17 control cases. They demonstrated that mean PSWE values were significantly greater in the DKD group (*p* < 0.001) and found a positive correlation between PSWE and albuminuria values [23].Additionally, our results are consistent with those of Maralescu et al., who demonstrated a strong correlation between histological grades of renal fibrosis and elastography-derived stiffness values in patients with chronic glomerulonephritis [24]. These findings highlight the clinical utility of PSWE in detecting structural kidney changes before overt functional impairment becomes apparent. Another study emphasize the utility of Shear wave elastography (SWE) which serve as a non-invasive tool to detect early renal involvement and monitor disease progression in Familial Mediterranean Fever patients [25]. 

Our research observed a positive correlation between RRI and PSWE values (p-values < 0.001). These findings indicate that RRI values increase, suggesting higher intrarenal resistance and PSWE values increase, reflecting greater renal stiffness and fibrotic activity. Additionally, we found strong positive correlations between periostin levels, RRI, and PSWE, reinforcing the link between fibrosis and renal dysfunction in DKD.The study emphasizes the unique value of combining periostin, RRI, and PSWE, which offers a robust, innovative and complementary markers for evaluating renal fibrosis. Moreover, it enables a more comprehensive and accurate evaluation of early detection and follow-up of DKD compared with conventional single-marker assessments.

The findings in our study are consistent with a study by Krishnan and Malik that involved 260 subjects (130 with DKD and 130 healthy controls), evaluating the correlation between RRI and PSWE. The study found that the mean RRI and PSWE was significantly greater in DKD [26]. Furthermore, a study by Baz et al. examined 26 diabetic patients and 26 healthy controls using PSWE and renal Doppler. This study reported significantly higher RRI and PSWE values in diabetic patients compared to controls across different renal zones (upper, middle, and lower), with p-values < 0.001 [27].

Our study demonstrated significant correlations between renal sonographic parameters (RRI and PSWE) and various clinical and laboratory variables. Notably, strong positive correlations were found between these sonographic parameters and clinical markers, including duration of diabetes, HbA1C, and BMI.

A study by Jinadu et al. noted that the duration of diabetes and HbA1C were associated with elevated RI in participants with DKD [28]. Furthermore, Diego et al. concluded that the relationship between RRI and albuminuria was influenced by increased BMI and obesity. This is consistent with literature suggesting that obesity exacerbates renal fibrosis through inflammatory and hemodynamic pathways [29].

## Conclusion

Periostin level, in conjunction with RRI and PSWE, serves as a novel and reliable markers of renal fibrosis. This integrative approach plays a crucial role in the early detection and monitoring of the progression of DKD. This combined analysis advances beyond single-parameter studies to enhance diagnostic precision of the extent of renal fibrosis. These enable timely intervention and more effective management of DKD.

### Limitations

This study has several limitations that should be acknowledged. Due to the relatively small sample size, the control group was not systematically matched to patients by age, sex, or BMI. Moreover, single-center design may limit the generalizability of the findings. Additionally, its cross-sectional nature restricts tracking patients over time, which is needed to determine the rate and pattern of transition between DKD stages in the same individuals; this is in contrast to longitudinal studies. Moreover, as both Doppler and elastography measurements are operator-dependent, some degree of measurement bias cannot be fully excluded, despite strict adherence to standardized acquisition protocols. Future prospective multicenter studies with larger and more diverse populations are warranted to confirm these observations and further delineate their clinical significance.

## Data Availability

This material has not been published previously, in whole or part, and is not under consideration for publication elsewhere. This paper has no tables or figures that would require permission to reprint.

## Abbreviations

DKD: Diabetic kidney disease
PSWE: Point shear wave elastography
RRI: Renal resistivity index
GFR: Glomerular filtration rate
ACR: Albumin-creatinine ratio
CKD: Chronic kidney disease
ROI: Regions of interest
BMI: Body mass index
HbA1c: Glycated hemoglobin (%)
