# Correction: Role of serum periostin level as profibrotic marker in conjunction with renal resistivity index and shear wave elastography in predicting renal fibrosis in diabetic nephropathy

**DOI:** 10.1186/s12882-026-05150-2

**Published:** 2026-06-25

**Authors:** Heba Mahmoud Ibrahim, Suldan A. Ali, Tarek A. Ramzy, Nehal H. Elsaid, Amr Mohamed Shaker

**Affiliations:** 1 Departments of Internal Medicine and Endocrinology, Kasr Al Aini Hospital, Cairo University, Cairo, Egypt; 2 Departments of Internal Medicine and Nephrology, Kasr Al Aini Hospital, Cairo University, Cairo, Egypt; 3 Department of Chemical Pathology, Kasr Al Aini Hospital, Cairo University, Cairo, Egypt

**Correction: BMC Nephrology (2025) 26:682**

10.1186/s12882-025-04612-3

In this article the author’s name “Heba Mahmoud Ibrahim” was incorrectly written as “Heba M. Ibrahim”. The original article has been corrected.

